# Band Alignment of Oxides by Learnable Structural-Descriptor-Aided Neural Network and Transfer Learning

**DOI:** 10.1021/jacs.3c13574

**Published:** 2024-03-28

**Authors:** Shin Kiyohara, Yoyo Hinuma, Fumiyasu Oba

**Affiliations:** † Laboratory for Materials and Structures, Institute of Innovative Research, Tokyo Institute of Technology, R3-7, 4259 Nagatsuta, Midori-ku, Yokohama 226-8501, Japan; ‡ Institute for Materials Research, Tohoku University, 2-2-1 Katahira, Aoba-ku, Sendai 980-8577, Japan; § Department of Energy and Environment, National Institute of Advanced Industrial Science and Technology (AIST), 1-8-31 Midorigaoka, Ikeda, Osaka 563-8577, Japan; ∥ MDX Research Center for Element Strategy, International Research Frontiers Initiative, Tokyo Institute of Technology, SE-6, 4259 Nagatsuta, Midori-ku, Yokohama 226-8501, Japan

## Abstract

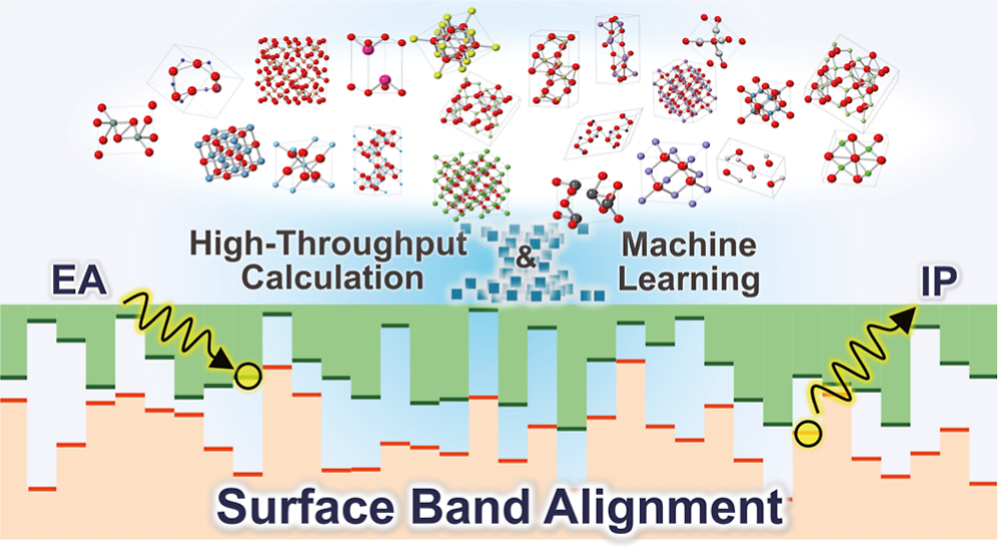

The band alignment of semiconductors, insulators, and dielectrics is relevant to diverse material properties and device structures utilizing their surfaces and interfaces. In particular, the ionization potential and electron affinity are fundamental quantities that describe surface-dependent band-edge positions with respect to the vacuum level. Their accurate and systematic determination, however, demands elaborate experiments or simulations for well-characterized surfaces. Here, we report machine learning for the band alignment of nonmetallic oxides using a high-throughput first-principles calculation data set containing about 3000 oxide surfaces. Our neural network accurately predicts the band positions for relaxed surfaces of binary oxides simply by using the information on bulk structures and surface termination planes. Moreover, we extend the model to naturally include multiple-cation effects and transfer it to ternary oxides. The present approach enables the band alignment of a vast number of solid surfaces, thereby opening the way to a systematic understanding and materials screening.

## Introduction

The ionization potential (IP) and electron affinity (EA) of a nonmetallic solid are defined as the energy levels of the valence band maximum (VBM) and conduction band minimum (CBM) with respect to the vacuum level, respectively. They show not only how easily an electron is released or accepted but also information about the relative band positions of materials. Such band alignments or lineups play crucial roles in our understanding, design, and development of materials and devices utilizing surfaces and heterointerfaces, including photocatalysts,^1−3^ photovoltaics,^4−6^ and all other heterostructured electronic and optoelectronic devices.^7−13^ The lattice mismatch, local atomic structure, and charge transfer should also be considered for depicting complete band alignments at heterointerfaces; nevertheless, IPs and EAs are fundamental information for designing interfacial functionalities in addition to surface-related properties.^7^

The IPs and EAs of solids involve contributions of surface dipole moments. Thus, they significantly depend on the atomic and electronic structures in the vicinity of the surfaces and therefore on the surface orientation and composition.^14−16^ Computationally, first-principles calculations have successfully quantified IPs and EAs.^15−23^ Separate calculations of bulk and surface systems are performed in a typical procedure for the evaluation of IPs and EAs. Bulk calculations offer VBM and CBM positions with respect to a reference level; surface calculations offer the differences between the vacuum and reference levels, which include the surface dipole contributions to electrostatic potentials. IPs and EAs are then obtained by the alignment of the bulk and surface reference levels.

However, an accurate and high-throughput evaluation of IPs and EAs is not straightforward because both processes are time-consuming. Density functional theory calculations using standard local and semilocal functionals produce large errors in the VBM and CBM positions, requiring us to use more elaborate and computationally demanding approaches, e.g., hybrid functionals including nonlocal exchange contributions and *GW* approximations based on many-body perturbation theory.^15−20,24,25^ Surfaces have both macroscopic and microscopic degrees of freedom, namely, the surface orientation as given by Miller indices and the location of the termination plane. Substantial reconstruction from ideal structures also takes place at some surfaces,^26−33^ which can significantly affect band positions as reported for perovskite oxides and TiO_2_.^32,33^ Therefore, even one material has a large variety of surface atomic structures, and systematic evaluation of their IPs and EAs requires huge amounts of computation.

Recently, machine learning has been widespread in materials science. Virtual screening based on machine learning is an efficient way to explore new materials with desired functions, where a surrogate model enables us to virtually predict material properties.^34−39^ In particular, theoretical calculations can generate comparatively large data sets that are prerequisites for constructing accurate surrogate models. Previous studies have reported successful materials screening for various properties by machine learning in combination with theoretical calculations.^35−38,40−44^

**Figure 1:**
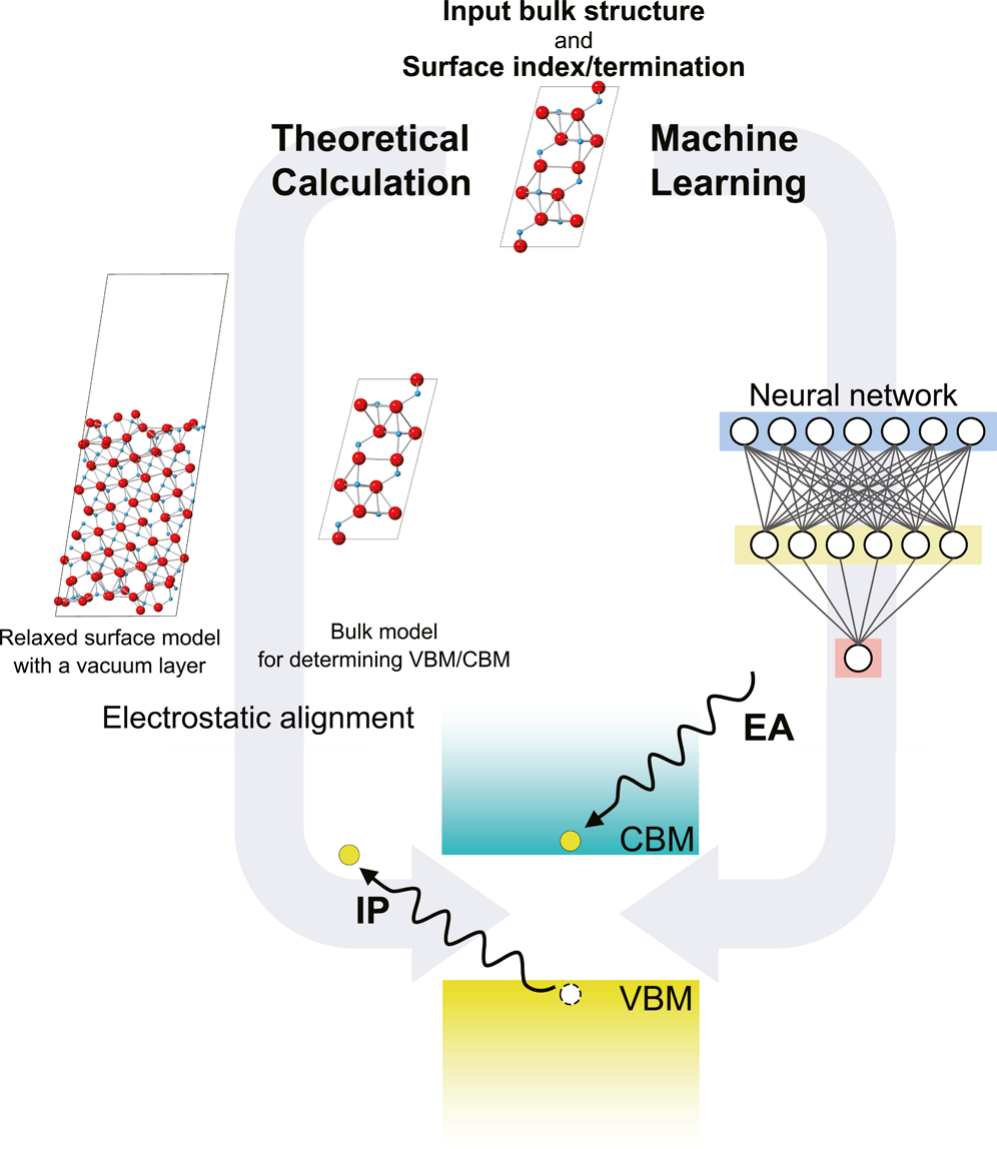
Schematic of prediction of IPs and EAs in nonmetallic solids by theoretical calculations and machine learning. The theoretical calculations from first principles typically use a combination of surface and bulk models to evaluate the energy difference between the vacuum level and the VBM (IP) or CBM (EA). Our ANN predicts the IPs and EAs of relaxed surfaces by simply inputting the information on the bulk crystal structure and the surface index and termination plane.

In this article, we present a regression model based on an artificial neural network (ANN) using the smooth overlap of atom positions (SOAPs)^45^ as input descriptors to predict the IPs and EAs of nonmetallic oxides (see Figure 1). First-principles calculations based on a non-self-consistent dielectric-dependent hybrid functional approach^21^ allow for the accurate and efficient evaluation of the IPs and EAs of about 3000 binary and ternary oxide surfaces. The ANN model constructed using this data set accurately predicts the IPs and EAs of binary oxides despite the model using the information before structural relaxation at surfaces as the input, namely, bulk crystal structures and surface termination planes of the oxides of interest. Moreover, we enable the ANN model to be applied to ternary oxides by developing “learnable” SOAPs, which can incorporate atom species varieties while keeping low descriptor dimensions.

## Results and Discussion

### High-Throughput First-Principles Calculations of IPs and EAs of Binary Oxides

**Figure 2:**
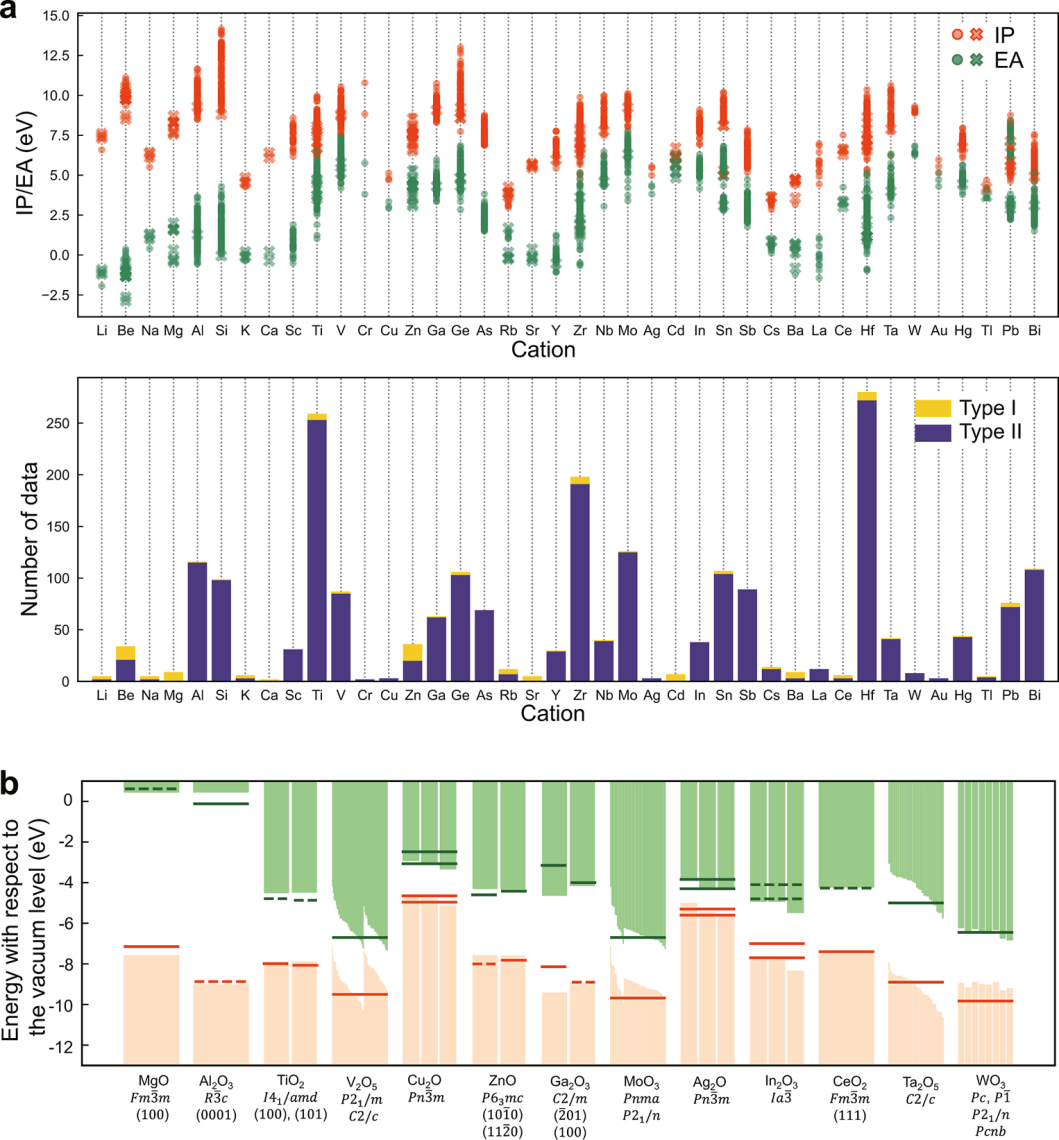
Distribution of theoretical IPs and EAs of binary oxides and comparison with experiments. (a) Upper panel shows the distribution of the IPs and EAs of the respective binary oxides. Orange and green dots are IPs and EAs, respectively. Cross and circle symbols are Tasker’s type I and II surfaces,^64^ respectively. The bottom panel is the number of surfaces, where yellow and dark blue bars are types I and II, respectively. (b) Theoretical IPs and EAs versus reported experimental values for selected binary oxides. The upper edges of the pale orange bars and the lower edges of the light green bars are calculated VBMs and CBMs with respect to the vacuum level (set at 0 eV), respectively. The orange and green solid lines are experimentally reported IPs and EAs, respectively; the dashed lines are derived by combining experimental IPs or EAs and experimental band gaps. The experimental data are taken from refs (70 and 71) for MgO, refs (72 and 73) for Al_2_O_3_, ref (74) for TiO_2_, ref (75) for V_2_O_5_, ref (76) for Cu_2_O, refs (77–79) for ZnO, refs (80 and 81) for Ga_2_O_3_, ref (82) for MoO_3_, refs (83 and 84) for Ag_2_O, refs (85 and 86) for In_2_O_3_, ref (87) for CeO_2_, ref (83) for Ta_2_O_5_, and ref (88) for WO_3_. The surface orientations have not been presented in the experimental reports for V_2_O_5_, Cu_2_O, MoO_3_, Ag_2_O, In_2_O_3_, Ta_2_O_5_, and WO_3_. Therefore, all theoretical IPs and EAs of binary oxides with the indicated space groups are depicted in the figure. Note that there are many types of surfaces for V_2_O_5_, MoO_3_, and Ta_2_O_5_, and the bars for each surface are extremely narrow.

Figure 2a shows distributions of the theoretical IPs and EAs in the binary oxide data set for 2195 nonpolar surfaces; all IP and EA values can be found in the Supporting Information. Here, we focus on nonpolar surfaces because the modeling of polar surfaces requires the consideration of system- and environment-dependent charge-compensation mechanisms^26,27,46^ and therefore their high-throughput calculations are not straightforward. The horizontal axis in Figure 2a is the constituent cation species, each of which contains various surfaces of several polymorphs. We find the tendency that the IP and EA values depend largely on the cation species, although the variety in the crystal and surface atomic structures leads to deviations of ∼ ±1 eV from their average values. The calculated IPs and EAs for selected surfaces are shown with available experimental values in Figure 2b. Although the IPs and EAs are, by nature, rather sensitive to the detailed structures and conditions of the experimentally investigated surfaces, reasonable agreement between the experiment and theory is recognized overall. The non-self-consistent dielectric-dependent hybrid functional approach taken here also well-reproduces the experimental IPs and EAs of group IV, III–V, and II–VI semiconductor surfaces.^21^ These comparisons with the experimental results demonstrate the accuracy of our high-throughput surface calculation data.

### Construction of ANN Models

**Figure 3:**
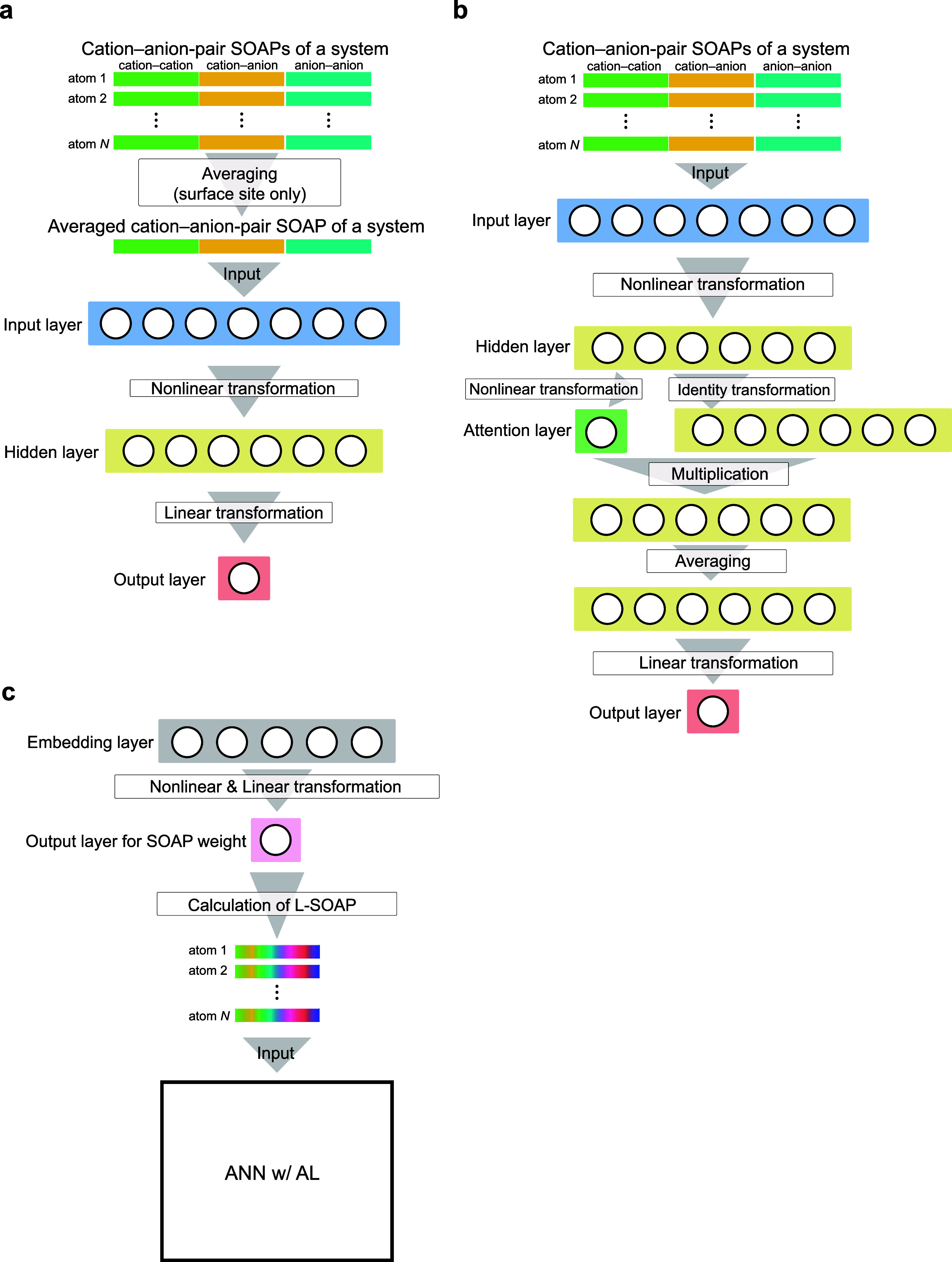
Architecture of ANNs. (a) Simple-ANN, (b) ANN w/AL, and (c) ANN w/L-SOAP. Each circle in the figure is a node where the input and output are scalars. Edges between nodes in two adjacent layers are fully connected but omitted for easy visualization.

**Figure 4:**
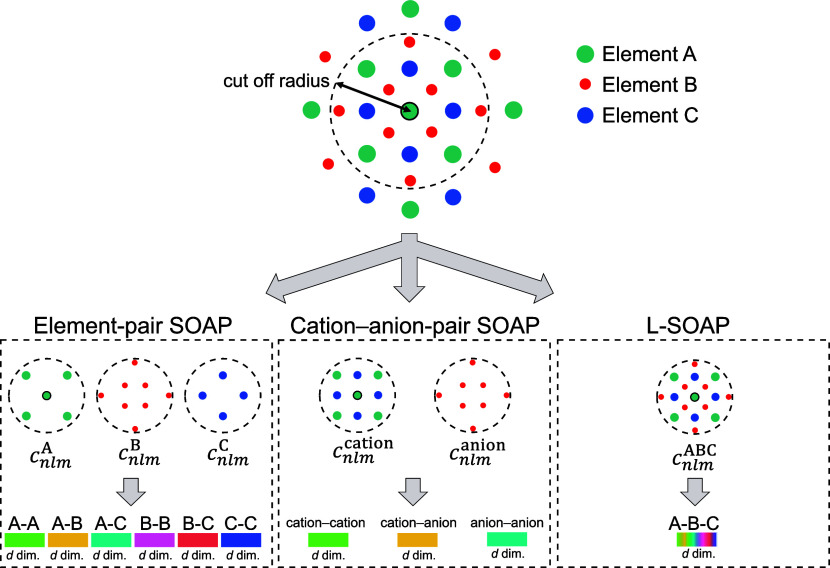
Schematic of conventional and learnable SOAP descriptors. The element-pair SOAPs (bottom left panel) are concatenations of the SOAPs of each elemental pair; the cation–anion-pair SOAPs (bottom center panel) are concatenations of three pairs, namely, cation–cation, cation–anion, and anion–anion combinations; and L-SOAPs (bottom right panel) have element-based learnable weights, which are automatically adjusted during ANN training.

Using the binary oxide data set, we constructed the ANN (hereinafter called simple-ANN) as shown in Figure 3a. The SOAP descriptors, which sophisticatedly include structural information such as the bond length, bond angle, and coordination number,^45^ are evaluated for unrelaxed surfaces and inputted in the first layer to describe the IPs and EAs at relaxed surfaces. The SOAPs in this study were preprocessed as follows. When multiple elements are included in a system, the concatenations of the SOAPs based on the elemental pairs (hereinafter called element-pair SOAPs) are generally used for describing atomic structures in a system^47^ as shown in the bottom left panel of Figure 4. Here, we consider 41 atom species simultaneously. Thus, the element-pair SOAPs have several tens of thousands of dimensions because SOAPs of a pair of elements have several tens to several hundreds of dimensions. To reduce the number of descriptor dimensions and prevent overfitting, we dealt with the 40 cation species as the same species when calculating SOAPs and oxygen as an anion, resulting in calculating the SOAPs of three pairs, namely, cation–cation, cation–anion, and anion–anion combinations (hereinafter called cation–anion-pair SOAPs), as shown in the bottom center panel of Figure 4. The cation–anion-pair SOAPs were calculated on each atom site in a system while the IP and EA need one-to-one correspondence with a system. Thus, we averaged the cation–anion-pair SOAPs in a system for inputs. Assuming that the information about the vicinity of the surfaces is critical to the IPs and EAs, we extracted the surface-region atoms by the scheme developed in ref (48) and averaged their SOAPs. More details of the architecture of the simple-ANN and SOAPs are described in “Machine Learning: SOAP Descriptors” and “Procedures for Regression” in Methods. The best combinations of *n*_max_ and *l*_max_, which are hyperparameters for SOAPs (see “Machine Learning: SOAP Descriptors” in Methods), are 3 and 7 based on root mean squared errors (RMSEs) and 5 and 3 based on mean absolute errors (MAEs), respectively (see Figure S1). Hereinafter, we show the results for *n*_max_ and *l*_max_ of 5 and 3, respectively; the other case is shown in Table S1. It should be noted that the results do not depend much on the combinations of *n*_max_ and *l*_max_.

**Figure 5:**
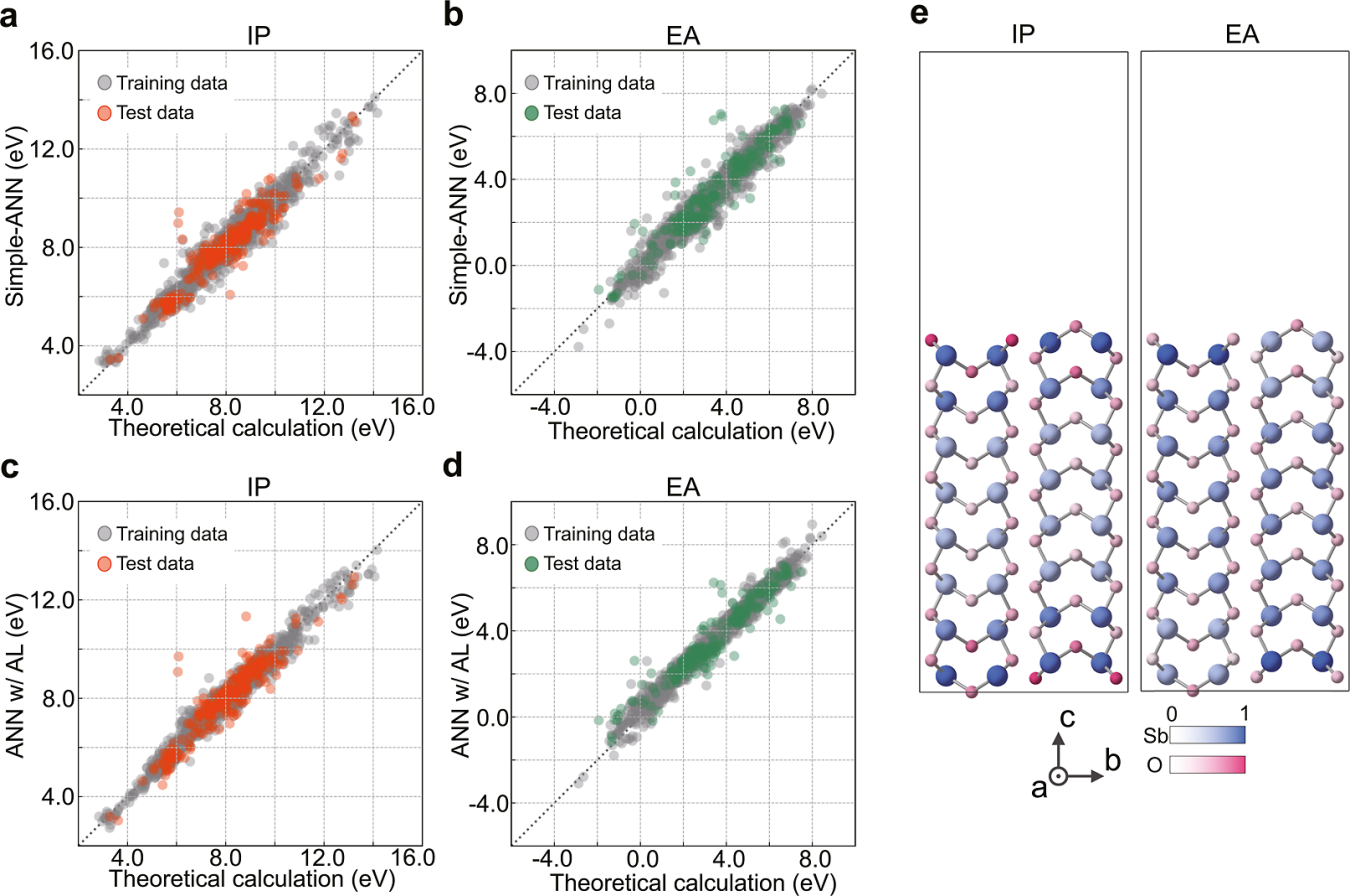
Theoretical and predicted IPs and EAs using simple-ANN and ANN w/AL. (a) IPs and (b) EAs obtained by first-principles calculations versus those predicted by the simple-ANN. (c) IPs and (d) EAs by first-principles calculations versus those predicted by the ANN w/AL. The orange or green and gray dots represent the test and training data, respectively. (e) Atom-site weights from the attention layer in the IP and EA prediction of a (001) surface of Sb_2_O_3_ whose space group is *Pccn* (index is 20 in Table S3). The frame indicates the surface supercell where the upper vacant region corresponds to the vacuum layer. Larger and smaller circles are the Sb and O atoms, respectively. The weights are represented by the shades of the atom colors: blue for Sb and pink for O. The weights are normalized so that the largest weight is one.

Figure 5a,b show the predicted IPs and EAs of the training data (gray dots) and test data (orange and green dots), respectively. Most of the data points are located near the diagonal lines, which means that the IPs and EAs predicted by the ANN are considerably close to the corresponding theoretical values from first-principles calculations. The coefficient of determination (*R*^2^), RMSE, and MAE of the test data are 0.90, 0.31, and 0.22 eV for the IPs and 0.90, 0.32, and 0.23 eV for the EAs, respectively. The high prediction accuracy indicates that the ANN model has learned to differentiate the cation species through structural information, even though elemental information is not explicitly given. In addition, the errors are much smaller than the aforementioned distributions of the IPs and EAs for each cation species in the data set, which indicates proper learning of the surface structure dependence. It is noteworthy that our ANN model can also learn and predict surface energies at the level of accuracy shown in Table S2, enabling us to screen out unstable or unreasonable surfaces without explicit first-principles calculations.

Although the simple-ANN model shows valuable prediction accuracy, it has a disadvantage; that is, it equally weights surface-region atoms in a system when averaging their SOAPs. To overcome this disadvantage and further enhance the prediction accuracy, we introduced an attention layer into our ANN architecture.^49,50^Figure 3b shows the ANN architecture with an attention layer (hereinafter called ANN w/AL). In contrast to the simple-ANN model, cation–anion-pair SOAPs of a system are inputted into the ANN without averaging and then averaged after passing through the inserted attention layer just before the output layer. The attention layer outputs a scalar value, and afterward, the value is input into a softmax function. Thus, each SOAP in a system has a weight, the sum of which is one. The transformed cation–anion-pair SOAPs are multiplied with the weights, averaged, and finally input into the output layer. As shown in Figures 5c,d, the prediction accuracy is evidently improved by introducing the attention layer, with an *R*^2^ of 0.90, RMSE of 0.29 eV, and MAE of 0.21 eV for the IPs, and 0.93, 0.27, and 0.19 eV, respectively, for the EAs.

Furthermore, the ANN w/AL can automatically estimate the magnitude of the impact of each atom site on the IPs and EAs. Figure 5e exemplifies this feature for the case of a (001) surface of Sb_2_O_3_ (space group: *Pccn*). The IP prediction clearly shows the higher importance of the antimony and oxygen atoms in the vicinity of the surface, while the halves of the antimony atoms on the surface are especially relevant to the EA. The weight profiles of some other binary oxides are shown in Figure S2. The profiles depend on the oxides and the IP or EA but have features common to the surfaces of the same oxides. Generally, the atoms on the second layers from the surfaces tend to have the smallest weights and the IPs put more importance on the surface atoms than the EAs.

### Extension of SOAPs and Application to Ternary Oxides

**Figure 6:**
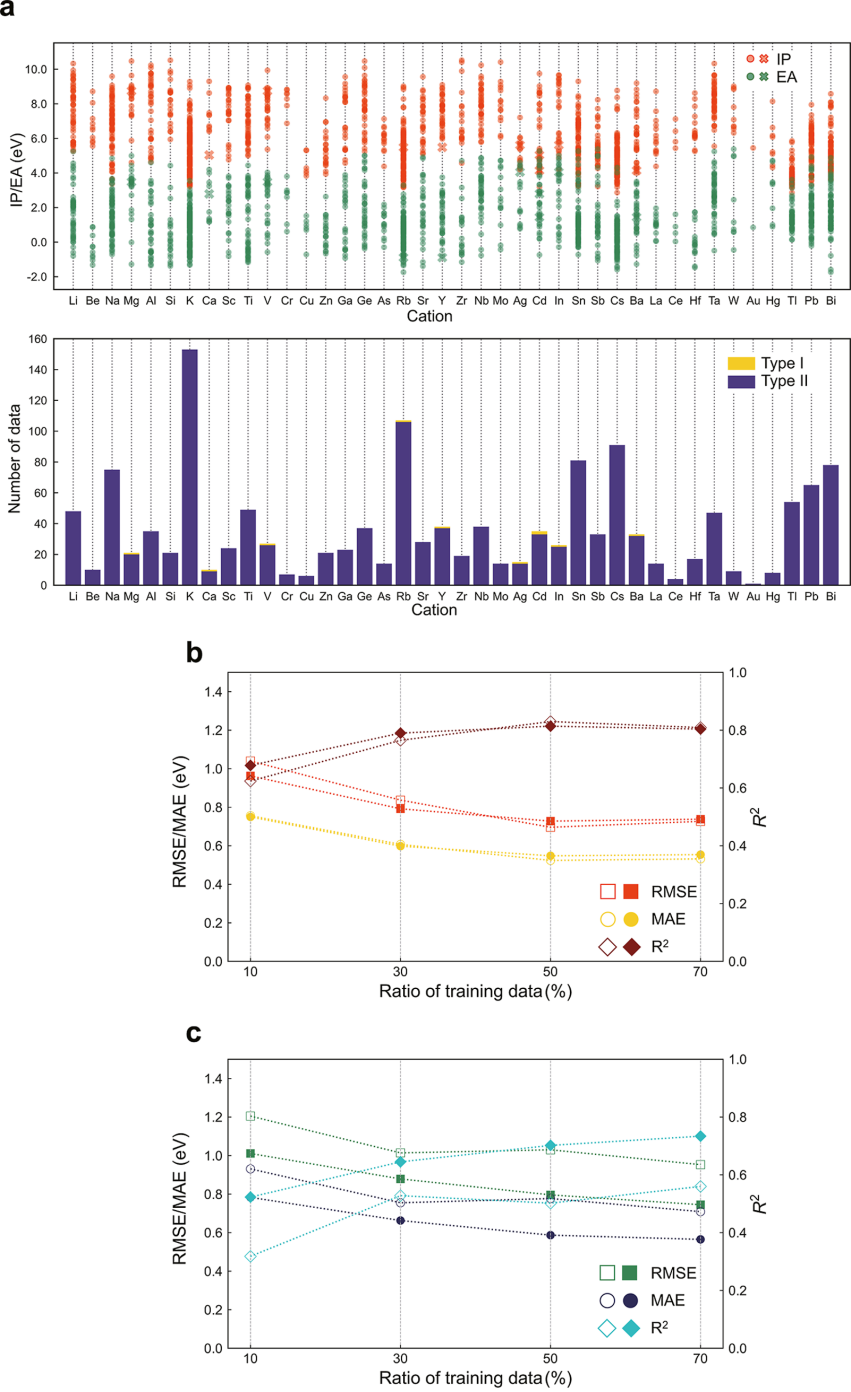
Distribution of theoretical IPs and EAs of ternary oxides and prediction accuracy of transfer learning. (a) Distribution of theoretical IPs and EAs. Ternary oxides include two cation species, and the same data points are shown at both cation species. The other details are the same as those for Figure 2a. (b,c) Prediction accuracy of transfer learning for IPs and EAs, respectively. The filled and open symbols are the results of the ANN w/L-SOAP and the ANN w/AL, respectively. The horizontal axis is the ratio of the ternary data for training to all ternary data.

Next, we focus on ternary systems. Figure 6a shows the theoretical IPs and EAs for 718 ternary oxide surfaces, with respect to the constituent cation species. The distributions of IPs and EAs are much larger than those in the binary systems partly because of the inclusion of multiple cation species in the ternary systems, as well as differences in the crystal and surface structures between the binary and ternary systems.

In the element-pair SOAPs, information about elements can be explicitly incorporated by concatenation of the SOAPs of each elemental pair (see bottom left panel of Figure 4), but this results in several thousands of dimensions in multispecies systems (see “Machine Learning: SOAP Descriptors” in Methods). The cation–anion-pair SOAPs have manageable dimensions but cannot differentiate cations in a system, which may be crucial for the ternary systems. Hence, we developed element-based learnable-weighted SOAPs (L-SOAPs), which have sufficient information about atomic species despite the considerably small number of dimensions. The details of the L-SOAPs are described in “Machine Learning: SOAP Descriptors” in Methods.

The new architecture of the ANN with L-SOAP (hereinafter called ANN w/L-SOAP) showed an *R*^2^ of 0.90, RMSE of 0.31 eV, and MAE of 0.23 eV for the IPs, and 0.91, 0.29, and 0.21 eV, respectively, for the EAs of the binary systems; this is comparable to the accuracy of the ANN w/AL. It is noteworthy that the L-SOAP has only one-third of the number of dimensions of the cation–anion-pair SOAP. The number of dimensions of element-pair SOAPs rapidly increases with the number of atomic species, whereas the L-SOAP keeps the same number of dimensions. This feature of the ANN w/L-SOAP model is a great advantage for dealing with multiple-element systems.

The trained model, however, cannot be directly applied to ternary systems, unlike the simple-ANN and the ANN w/AL models, because multiple-cation effects (eq 10 in Methods) are not learned from the binary systems. Thus, we trained the models using the binary oxide data set and afterward retrained them using the ternary oxide data set. In other words, we performed transfer learning from the binary systems to the ternary systems, where all the learning parameters in the ANN w/AL were retrained using the ternary oxide data set. The ratio of the ternary data for training to all ternary data was set at 10, 30, 50, and 70%, where the residues were used as test data. The effectiveness of “transferring” is shown in Figure S3. A clear improvement in the prediction accuracy is found for transfer learning compared with learning from scratch.

Figure 6b shows the performance of the IP prediction with an *n*_max_ of 5 and *l*_max_ of 3; see Figure S4 for the parity plots and Figure S5 for the case of an *n*_max_ of 3 and *l*_max_ of 7. For comparison, the results of the transferred ANN w/AL are included in Figure 6. The ANN w/L-SOAP and the ANN w/AL exhibit almost the same levels of performance in all the ratios of training and test data. The IPs relate to the VBMs, in which oxygen 2p-orbitals are the major components in most binary oxides. This fact also applies to the ternary oxides although the weak multiple-cation effects on the VBMs exist. Because the ANN w/L-SOAP mainly learns cation–cation interactions here, both of the ANNs show similar performances regarding the IPs.

We now move on to the prediction of the EAs. The main components of the CBMs are cation orbitals that are system-dependent. Because an accurate prediction of the EAs requires learning such CBM characters, one expects that the ANN w/AL, which cannot explicitly incorporate the effects of the variety of cations, would be inaccurate. Indeed, the ANN w/AL shows considerably poor accuracy when the ratio of the training data is 10% (Figure 6c). On the other hand, the ANN w/L-SOAP is clearly more accurate at the same training/test data ratio. Surprisingly, the performance of the ANN w/L-SOAP at the 10% ratio is almost equal to that of the ANN w/AL at the 70% ratio, and the performance at the 70% ratio is comparable to that of the IPs, indicating the successful incorporation of the multiple-cation effects during training. Thus, the ANN w/L-SOAP in combination with transfer learning makes accurate and systematic prediction of both IPs and EAs of ternary systems feasible.

## Conclusions

Our high-throughput first-principles calculations based on the non-self-consistent dielectric-dependent hybrid functional approach have enabled the construction of a large data set of IPs and EAs for 2195 binary and 718 ternary oxide surfaces. The accuracy of the calculated IPs and EAs has been confirmed by comparison with available experimental values for the selected binary oxides as shown in Figure 2b. Using the data set, we constructed the simple-ANN model to predict the IPs and EAs of the binary oxides. The cation–anion-pair SOAPs were used to describe surface atomic structures before structural relaxation, by simply inputting the crystal structures and surface termination planes. The IPs and EAs of relaxed surfaces were accurately predicted using the SOAP structural descriptors even though elemental information was not explicitly given. Furthermore, introducing an attention layer to the simple-ANN model allows for automatically determining relevant atoms in surface regions and enhancing the prediction performance. This feature of the attention layer can collaterally help us to analyze chemical and physical origins of target properties. Finally, we extended the SOAPs to transfer the model to the ternary oxides by introducing the element-based learnable weights to the calculation of the SOAPs and by connecting this process to the ANN w/AL. This transfer learning model based on the ANN w/L-SOAP was able to incorporate the small effects of the cations on the VBMs with the small data set in the IP prediction. Moreover, apparent improvement was demonstrated regarding the EA prediction, indicating that diverse cation effects on the CBMs were well incorporated.

The ANN w/L-SOAP developed here enables the band alignment of a vast number of metal oxide surfaces, which is fundamental to the design of various materials and devices. Moreover, it is not restricted to the prediction of surface properties of metal oxides but can be naturally applied to other multicomponent systems and other properties in the same way as crystal graph convolutional neural networks.^38^ Thus, we believe that our new model paves the way for the high-throughput prediction of diverse materials and properties.

## Methods

### Screening of First-Principles Calculation Data: Binary Oxides

The binary oxides considered in this work are listed in Table S3. The table contains 134 nonmetallic oxides whose band structures are formally constructed by fully occupied or empty atomic orbitals, including 41 types of atom species. To cover a wide variety of stable and metastable polymorphs, experimentally determined crystal structures of binary oxides were used as prototypes, and the cations of the prototypes were substituted with isovalent cations. The details of the cation substitution procedures are described in ref (51).

We excluded the oxides without the special isometry that nonpolar slabs should have.^52^ In addition, we screened out binary oxides with rather narrow theoretical band gaps using criteria of band gaps smaller than 0.1 eV and ion-clamped static dielectric constants larger than 20, resulting in the 127 binary oxides with cation species other than those in the parentheses in Table S3. Then, we generated 3383 nonpolar surface models for the 127 binary oxides with relatively small Miller indices as listed in Table S3. To exclude unreasonable surface structures, we selected surfaces that were smoothly optimized during first-principles calculations and those with surface energies higher than 0 J/m^2^. In addition, we excluded the outliers in surface energy, which did not fulfill the following inequality
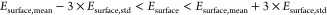
1where *E*_surface_ is the surface energy of the objective surface, and *E*_surface,mean_ and *E*_surface,std_ are the mean and standard deviations of the surface energies, respectively, in which the surfaces are generated from the common bulk system to the objective surface, resulting in the data set with 2195 binary oxide surfaces for machine learning.

### Ternary Oxides

We screened and collected ternary oxide data from the Materials Project database^53^ in the following manner: (1) extract the most stable binary oxides of A and B, where A and B are any of the 40 cation elements; (2) draw a convex hull of the formation energies of A–B ternary oxides with respect to the A binary oxide and the B binary oxide; (3) select the ternary oxides on the convex hull; and (4) exclude the ternary oxides without the special isometry and with band gaps smaller than 0.1 eV. We then evaluated the band gaps using non-self-consistent dielectric-dependent hybrid functional calculations and screened the ternary oxides in the same way as the binary oxides. We obtained 344 ternary oxides through these processes. Then, we made the nonpolar surface models, where the maximum Miller indices were set to be 2. These surfaces were screened in the same way as the binary cases, resulting in 718 ternary oxide surface data.

### First-Principles Calculations of Band Positions

IPs and EAs are estimated as follows^16^
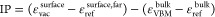
2
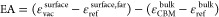
3where  and  are the vacuum level and the electrostatic reference level in the bulk-like region far from the surface, respectively. These quantities are obtained by calculations using surface supercells, each of which is composed of a slab of an oxide and a vacuum layer. , , and  are the VBM, CBM, and electrostatic reference levels in the corresponding bulk model, respectively. These three values are obtained by calculations using bulk primitive cells.

### Computational Procedures for Bulk Systems

The calculations were performed using the projector augmented-wave (PAW) method^54^ as implemented in the Vienna *abinitio* simulation package (VASP).^55,56^ The  and  were calculated by the approach reported in ref (21). In this scheme, the nonlocal exchange mixing parameter in the full-range hybrid functional of the Perdew–Burke–Ernzerhof (PBE0) form^57,58^ is set to be the inverse of the ion-clamped static dielectric constant of the system.^59,60^ The PBE functional tuned for solids (PBEsol)^61^ was used for the semilocal part of the hybrid functional. Non-self-consistent hybrid functional calculations^21,62^ were performed on top of PBEsol with Hubbard *U* corrections to localized orbitals^63^ to simultaneously attain low computational cost and high accuracy. This approach is particularly advantageous in the high-throughput evaluation of IPs and EAs as it allows for the direct alignment of the bulk hybrid functional eigenvalues with the vacuum levels in surface supercells obtained using PBEsol(+*U*) through the common electrostatic reference levels.^21^ For the reference level , we used the averaged local potentials at the oxygen sites.

PBEsol(+*U*) was used for obtaining optimized structures, static dielectric constants, and wave functions for the non-self-consistent dielectric-dependent hybrid functional calculations. The means of the diagonal elements of the ion-clamped static dielectric tensors obtained using the random phase approximation were taken to determine the values of the nonlocal exchange mixing parameter for respective systems.

### Computational Procedures for Surfaces

We focus on high-throughput calculations of nonpolar surfaces in this study because specific treatments are necessary for modeling polar surfaces as mentioned above. We prepared slab models for nonpolar surfaces of Tasker’s types I and II^64^ for the binary and ternary oxides. Their maximum Miller indices were set to be those listed in Table S3 for binary oxides and 2 for ternary oxides. Each slab model has a slab layer thicker than 20 Å and a vacuum layer thicker than 15 Å, which are large enough for the electrostatic potential to converge sufficiently.^21^ The convergence test results of IPs, EAs, and surface energies with respect to the vacuum and slab layer thicknesses are shown for selected systems in Figure S6. The slab models were automatically created using the code developed in ref (52). The surfaces were structurally relaxed with their lattice constants fixed by using PBEsol(+*U*).  was estimated as the electrostatic potential at the midpoint of the vacuum layer, and  was taken to be the average of the local potentials of the oxygen sites around the midpoint of the slab layer.

### Computational Details

The PAW data set used in the calculations is detailed in Table S4. The plane-wave cutoff energy was set to 520 eV for the bulk structure optimization, including lattice parameter relaxation, and 400 eV for the other calculations with the lattice parameters fixed. The *k*-point mesh spacings for structure optimization were set to be smaller than 0.2 Å^–1^ and reduced to be less than 0.1 Å^–1^ in the calculations of the ion-clamped static dielectric tensors. The band path for estimating the VBM and CBM of each bulk system was generated using SeeK-path.^65,66^ Hubbard *U* corrections were applied to localized orbitals by using the parameters listed in Table S4.

### Machine Learning: SOAP Descriptors

For input descriptors to describe surface atomic configurations, we employed SOAPs, which are often used to construct machine learning potentials and analyze complicated systems.^67,68^ In SOAPs, the positions of atoms labeled with *Z* (usually atomic species) are smeared with three-dimensional Gaussian functions as
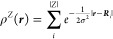
4where ***R***_*i*_ is the position of the *i*-th atom surrounding ***r***. |*Z*| is the number of atoms labeled *Z* within the cutoff radius and σ is a standard deviation of the Gaussian function. The summation of the smeared atom positions is then expanded by linear combinations of radial basis functions with a cutoff radius and spherical harmonics considering the local point of interest. The coefficients are defined as
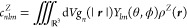
5where *g*_*n*_ and *Y*_*lm*_ are radial basis functions and spherical harmonics, respectively. The former generally depends on location ***r*** from the center and the latter on polar angle θ and azimuth ϕ in the polar coordinates around the center. *n* is the degree of the radial basis function; *l* and *m* are the degree and order of the spherical harmonics, respectively. The partial power spectrum vector is defined as
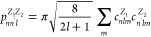
6where *Z*_1_ and *Z*_2_ are the symbols of elements. The element-pair and cation–anion-pair SOAPs were obtained on the basis of these procedures by using the DScribe code.^47^

In the L-SOAP, atomic positions are not separately considered for every atom species, unlike the usual SOAP procedure. Instead, we weighted the broadened atomic positions in eq 4 on every atom as
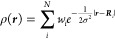
7where *w*_*i*_ is its weight and *N* is the number of atoms within the cutoff radius. The bottom right panel of Figure 4 shows the schematic of the L-SOAP. Here, we replaced the Gaussian function in eq 7 with the delta function for easy calculation, resulting in changing the operation of integration in eq 5 to a summation as
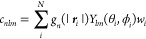
8

We connected the L-SOAPs to the ANN w/AL (see Figure 3c), which enables the model to learn the weights *w*_*i*_ in eq 8 during training. Atomic species described by one-hot vectors are encoded at an embedding layer to atomic feature vectors as
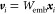
9where ***x***_*i*_ is a one-hot vector of an *i*-th atom in a system for describing its atomic species. *W*_emb_ is a weight matrix for encoding the one-hot vectors into the feature vectors ***v***_*i*_. Here, the dimensions of the feature vectors were set to 32. Then, the weights for the L-SOAPs in eq 8 are calculated as
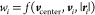
10where ***v***_center_ is the atomic feature vector of the centered atom and ***v***_*i*_ is that of the atom located at |***r***_*i*_| from the center. The function *f* is a simple neural network with two hidden layers. The coefficients and SOAPs are then calculated according to eqs 6 and 8. The rest of the processes are the same as those for the ANN w/AL.

SOAPs are typically inputted into a kernel function; however, we used nonkernel SOAPs, namely, the partial power spectrum vector in this study. The maxima of *n* (*n*_max_) and *l* (*l*_max_) for the radial basis functions and spherical harmonics were surveyed in combinations 3, 5, and 7. The cutoff radius for the radial basis functions was fixed to 7.0 Å and the standard deviation of the Gaussian function in eqs 4 and 7 was set at 1.0 Å. The relatively large cutoff radius was used to include the surface effects in the SOAPs for atoms on layers below the surfaces, thereby improving prediction accuracy. For instance, a smaller cutoff radius of 5.0 Å in the Simple-NN for the IP prediction shows an MAE of 0.38 eV for the test data, which is clearly less accurate than the case of 7.0 Å, 0.23 eV.

### Procedures for Regression

Among various regression techniques, we selected ANNs for predicting IPs and EAs in this study because they offer flexible architecture, automatic extraction of relevant surface-region atoms, as described in Results and Discussion, and transferability to other systems.

The simple-ANN shown in Figure 3a has a typical architecture with input, hidden, and output layers. The input vectors are averaged SOAPs of a system. Its hyperparameters, e.g., the number of hidden layers, are listed in Table S5. The rectified linear unit was used as an activation function, and the dropout rate was fixed at 0.5 in hidden layers that were not linked with the output layer.

The data sets used in the regression were divided into training, validation, and test data sets with a ratio of 8:1:1, where we did not care about the distribution of the cation species in respective data sets. Fivefold cross validation using the validation data sets was performed for tuning the hyperparameters of the ANNs. We used backpropagation based on the Adam scheme^69^ to optimize all the learning parameters in the network, thereby minimizing the mean squared errors between the model outputs and the training targets. The learning rate for the optimization was 0.001, and the maximum epoch was 200. The training was terminated when the prediction accuracy of the validation data was converged to be almost constant as exemplified by the IP learning curve for the simple-NN in Figure S7. All of the training converged well within 200 epochs.

## Supporting Information Available

RMSE and MAE of all the considered combinations of the SOAP hyperparameters; profile of the weights from the attention layer; transfer learning versus learning from scratch; theoretical and predicted IPs and EAs for the ternary data sets; convergence of IPs, EAs, and surface energies; learning curve for IPs; prediction accuracies; 134 prototypical binary oxides and surface orientation; PAW datasets and Hubbard *U* parameters; and hyperparameters of the simple-ANN (PDF)
Theoretical IPs and EAs of the 2195 binary and 718 ternary oxide surfaces and related properties. Database contains the index, system (binary or ternary), chemical formula, space group number, Miller index, surface energy, IP, EA, and band gap (bulk) (XLSX)
Supercells before and after structural optimization for the 2195 binary and 718 ternary oxide surfaces (ZIP)
