# Supplementary material for: Band Alignment of Oxides by Learnable Structural-Descriptor-Aided Neural Network and Transfer Learning
Source: J Am Chem Soc. 2024 Mar 28;146(14):9697–708. doi: 10.1021/jacs.3c13574 (PMC11009958; doi:10.1021/jacs.3c13574)
Supplement: Supplementary file 1 — ja3c13574_si_001.pdf [file ja3c13574_si_001.pdf]

Supporting Information: Band Alignment of Oxides by Learnable Structural-Descriptor-Aided Neural Network and Transfer Learning

Shin Kiyohara,[*,†,‡] Yoyo Hinuma,[§] and Fumiyasu Oba[**,†,⊥]

[†]Laboratory for Materials and Structures, Institute of Innovative Research, Tokyo Institute of Technology, R3-7, 4259 Nagatsuta, Midori-ku, Yokohama 226-8501, Japan

[‡]Institute for Materials Research, Tohoku University, 2-2-1 Katahira, Aoba-ku, Sendai 980-8577, Japan

[§]Department of Energy and Environment, National Institute of Advanced Industrial Science and Technology (AIST), 1-8-31 Midorigaoka, Ikeda, Osaka 563-8577, Japan

[⊥]MDX Research Center for Element Strategy, International Research Frontiers Initiative, Tokyo Institute of Technology, SE-6, 4259 Nagatsuta, Midori-ku, Yokohama 226-8501, Japan

* Email: shin.kiyohara.d6@tohoku.ac.jp
** Email: oba@msl.titech.ac.jp

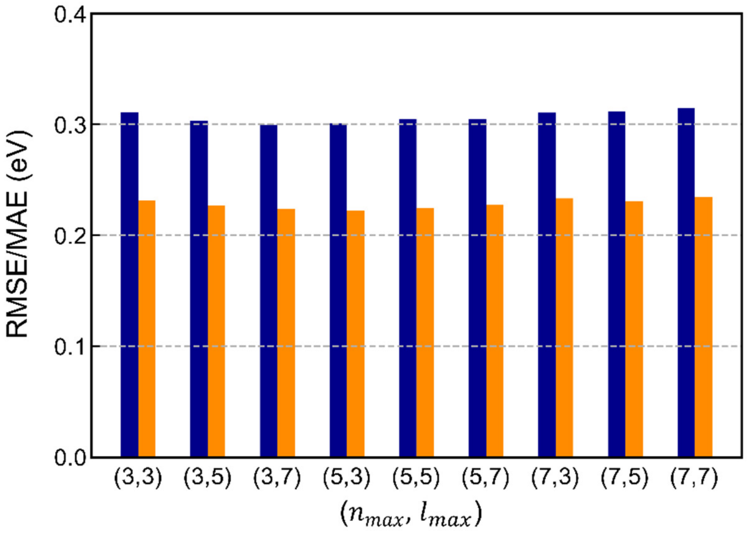

Figure S1. RMSE and MAE of all the considered combinations of n_{max} and l_{max} in SOAP descriptors. The dark blue and orange bars are RMSEs and MAEs, respectively.

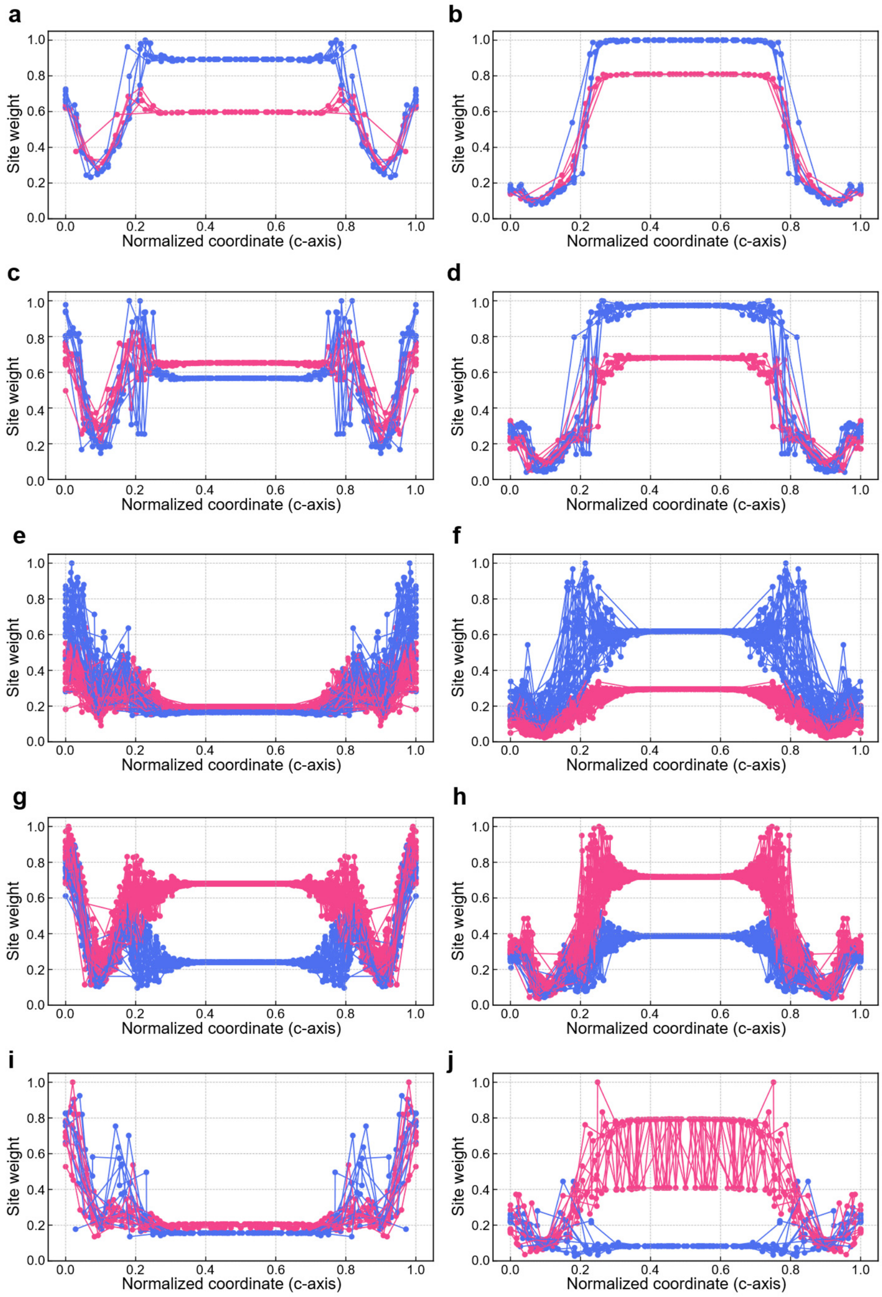

Figure S2. Profile of the weights from the attention layer. (a),(c),(e),(g),(i) IPs of surfaces of Li_2O with index 3, ZnO with index 6, HgO with index 9, Al_2O_3 with index 15, and Y_2O_3 with index 16 in Table S3 and (b),(d),(f),(h),(j) their EAs, respectively. The horizontal axis is a normalized atom coordinate in the direction perpendicular to the surface, where the largest coordinate in each slab model is set to one. Blue and pink dots are cations and anions (oxide ions), respectively. The weights in the same slab are linked by the line. The weights in the same oxides are normalized so that the largest weight inputted into a softmax function in the attention layer is one.

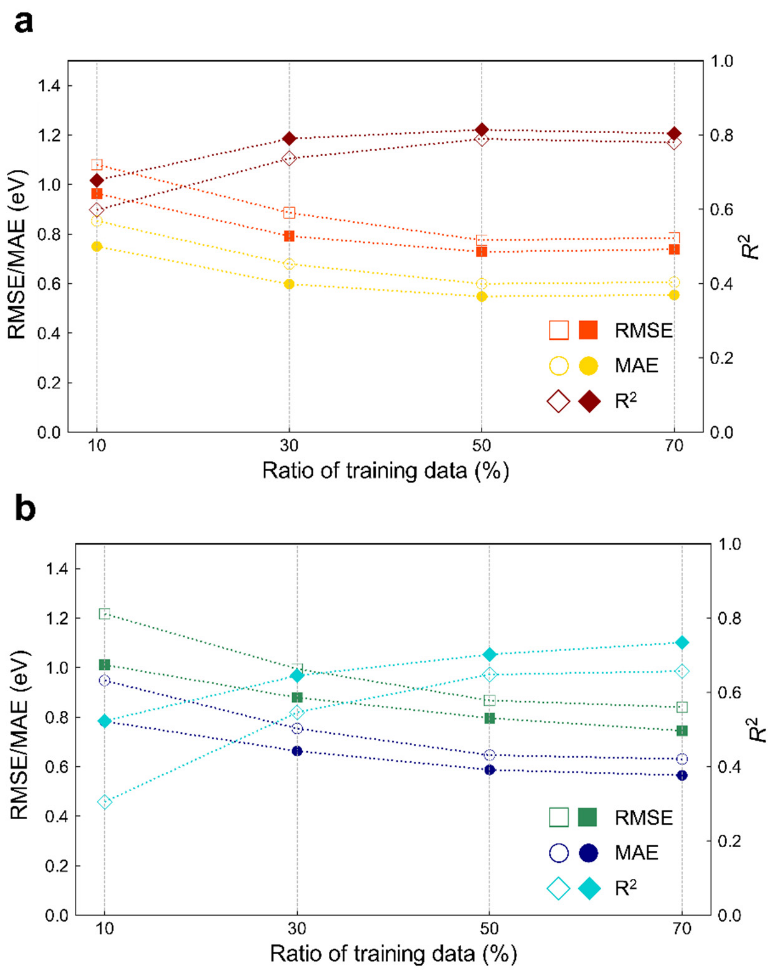

Figure S3. Transfer learning versus learning from scratch when n_{max} is 5 and l_{max} is 3. (a) IPs and (b) EAs. The filled and open symbols are the results of the transfer learning and learning from scratch using the ANN w/ L-SOAP, respectively. The horizontal axis shows the ratio of the ternary data for training to all ternary data.

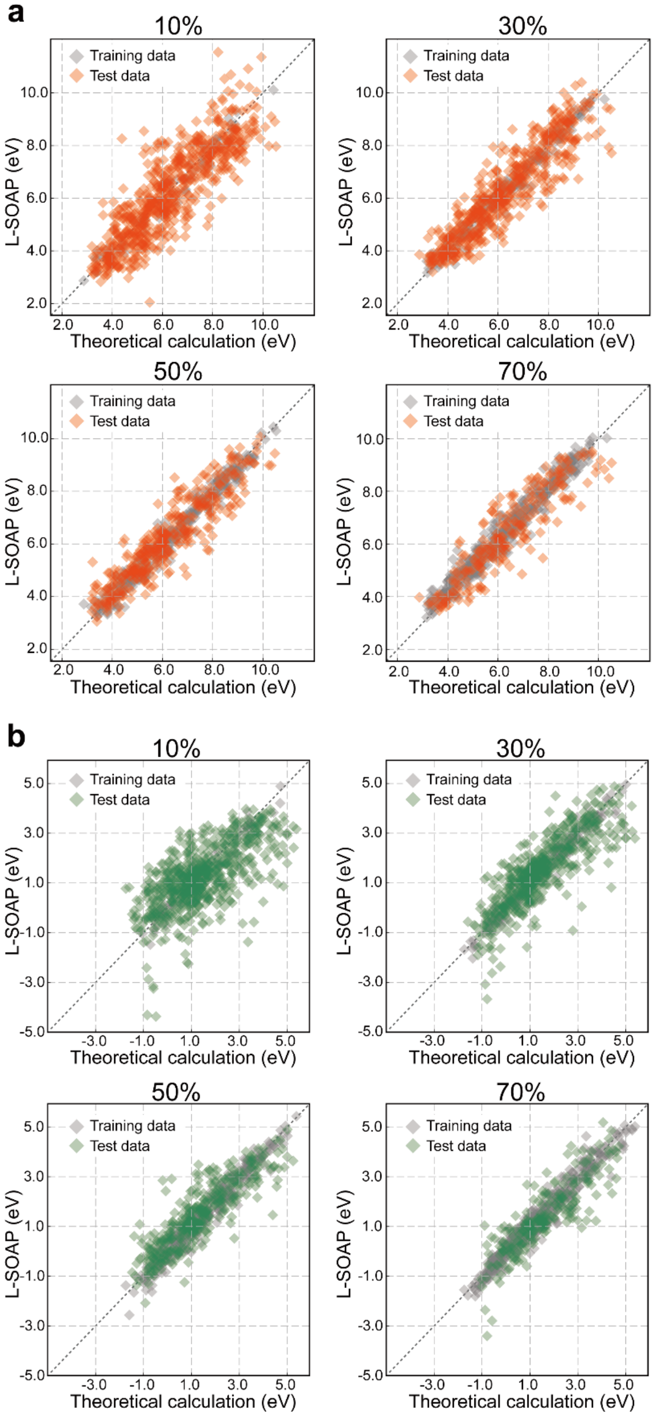

Figure S4. Theoretical and predicted IPs and EAs for the ternary datasets using the ANN w/ L-SOAP when n_{max} is 5 and l_{max} is 3. (a) IPs and (b) EAs. The proportion of the training to the total data is indicated above each panel.

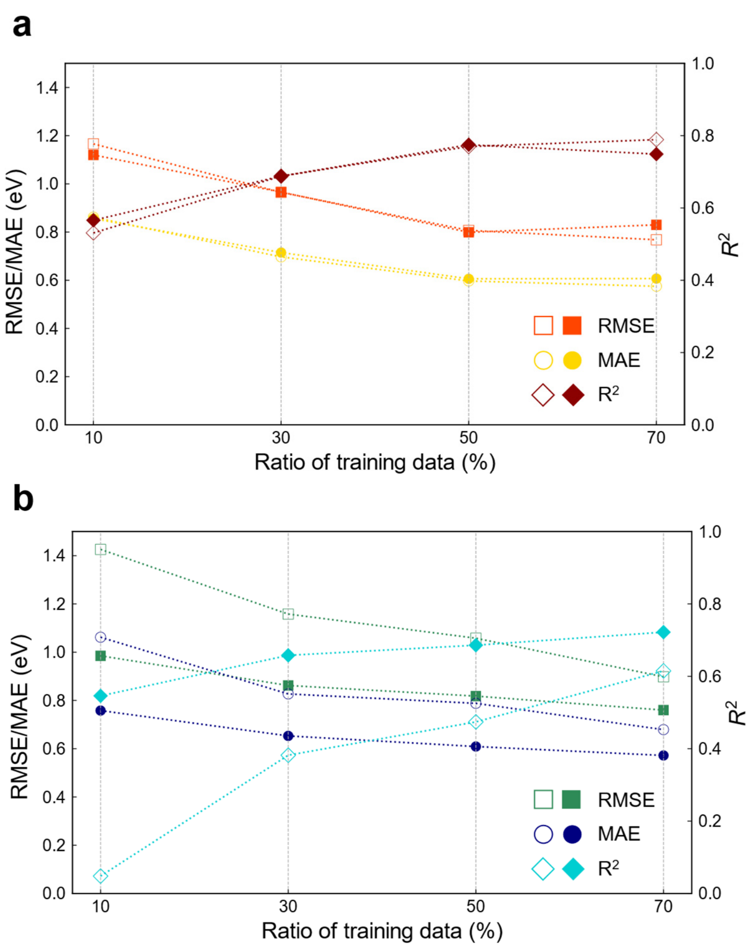

Figure S5. Prediction accuracy of the ANN w/ L-SOAP when n_{max} is 3 and l_{max} is 7. (a) IPs and (b) EAs. The filled and open symbols are the results of the ANN w/ L-SOAP and the ANN w/ AL, respectively. The horizontal axis shows the ratio of the ternary data for training to all ternary data.

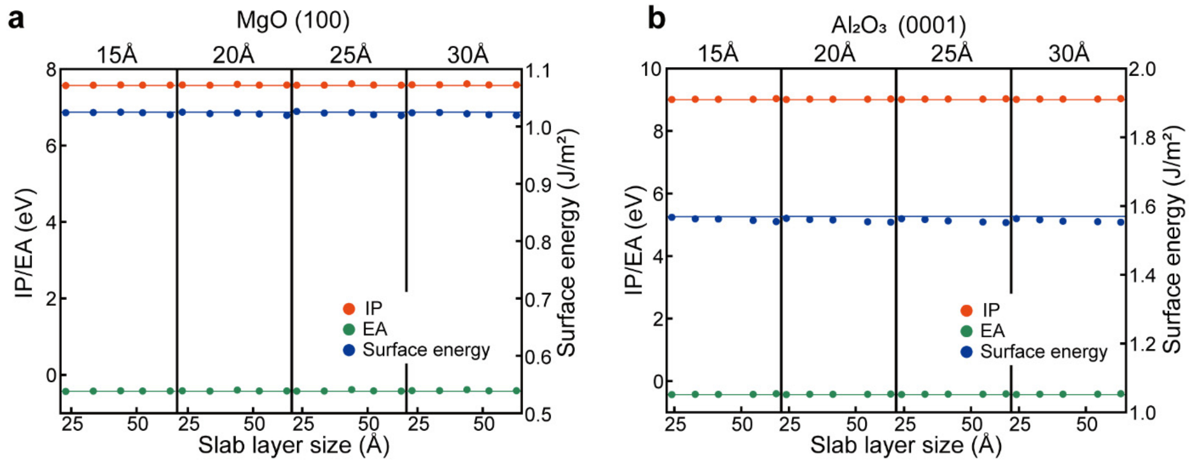

Figure S6. Convergence of IPs, EAs, and surface energies with respect to the thicknesses of the vacuum and slab layers. (a) (100) surface of MgO whose space group is $Fm\bar{3}m$. (b) (0001) surface of Al_2O_3 whose space group is $R\bar{3}c$. The numbers above respective panels are the vacuum layer sizes. The horizontal lines represent the IPs, EAs, and surface energies in the datasets used in this work.

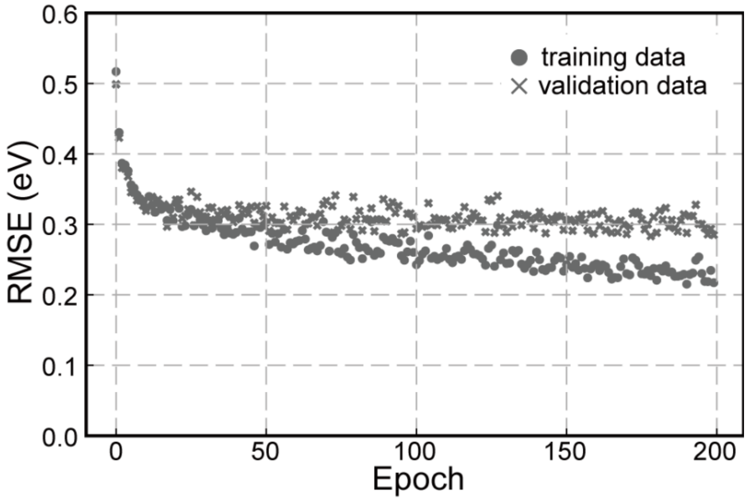

Figure S7. Learning curve for IPs when using the simple-NN.

Table S1. Prediction accuracy of the three types of ANN of (n_{max}, l_{max}) = (3,7) and (5,3) for the IP and EA. The slash-separated values from left to right are R^2, RMSE, and MAE. The unit of RMSE and MAE is eV.

	(n_{max}, l_{max}) = (3,7)		(n_{max}, l_{max}) = (5,3)	
	IP	EA	IP	EA
Simple-ANN	0.89/ 0.31/ 0.23	0.90/ 0.31/ 0.23	0.90/ 0.31/ 0.22	0.90/ 0.32/ 0.23
ANN w/ AL	0.91/ 0.28/ 0.20	0.92/ 0.28/ 0.20	0.90/ 0.29/ 0.21	0.93/ 0.27/ 0.19
ANN w/ L-SOAP	0.89/ 0.32/ 0.23	0.91/ 0.30/ 0.22	0.90/ 0.31/ 0.23	0.91/ 0.29/ 0.21

Table S2. Prediction accuracy of the three types of ANN of (n_{max}, l_{max}) = (3,7) and (5,3) for the surface energy. The slash-separated values from left to right are R^2, RMSE, and MAE. The unit of RMSE and MAE is J/m^2.

	(n_{max}, l_{max}) = (3,7)	(n_{max}, l_{max}) = (5,3)
Simple-ANN	0.76/ 0.47/ 0.33	0.76/ 0.48/ 0.34
ANN w/ AL	0.83/ 0.41/ 0.29	0.79/ 0.44/ 0.31
ANN w/ L-SOAP	0.73/ 0.51/ 0.35	0.76/ 0.47/ 0.33

Table S3. 134 prototypical binary oxides and surface orientation. The space groups of prototype crystal structures, the elements considered as cations in binary oxides, and the maximum Miller indices for creating surface models are indicated. The oxides with the cation species in the parentheses have been screened out by the procedures given in 'Screening of First-Principles Calculation Data: Binary Oxides' in 'METHODS'.

Index	Space group	Cation	Maximum Miller indices
1	$P\bar{3}m$	Rb, Cs, Tl	2, 2, 2
2	$R\bar{3}m$	Cs, (Tl)	2, 2
3	$Fm\bar{3}m$	Li, Na, K, Rb	3, 3, 3, 3
4	$Pn\bar{3}m$	Cu, Ag, Au	3, 3, 3
5	$Fm\bar{3}m$	Be, Mg, Ca, Zn, Sr, Cd, (Sn), Ba	2, 2, 2, 2, 2, 2, 2, 2
6	$P6_3mc$	Be, Mg, Zn, Cd, Sn	3, 2, 3, 3, 2, 3
7	$F\bar{4}3m$	Be, Mg, Ca, Zn, Sr, (Cd), (Sn), Ba	1, 1, 1, 1, 1, 1, 1, 1
8	$P4_2/mnm$	Be, Zn	2, 2
9	$Pnma$	Hg	2
10	$P3_121$	Hg	4
11	$Pbcm$	Sn, Pb	2, 2
12	$P4/nmm$	Pb	2
13	$P6_3/mmc$	Ba	4
14	$Ia\bar{3}$	Al, Sc, Ga, Y, In, La, Bi	1, 1, 1, 1, 2, 2, 2
15	$R\bar{3}c$	Al, Sc, Ga, Y, In, La	4, 4, 4, 4, 4, 4
16	$P\bar{3}m1$	Y, La, Bi	2, 2, 2
17	$C2/m$	Al, Ga	4, 2
18	$P2_1/n$	As	2
19	$Fd\bar{3}m$	As, Sb	3, 3
20	$Pccn$	As, Sb, Bi	2, 2, 2
21	$P2_1/c$	Sb, Bi	2, 2
22	$P\bar{4}2_1c$	Sb, Bi	2, 2
23	$P6_3mc$	Al, Ga, In	2, 2, 2
24	$P4_2/mnm$	Si, Ti, Ge, Sn, Hf	2, 2, 2, 2, 2
25	$I4_1/amd$	Ti	3
26	$Pbca$	Ti, Hf	2, 2
27	$Pbca$	Zr, Hf, (Pb)	2, 2, 2
28	$P2_1/\mathrm{c}$	Ti, Zr, Hf, (Pb)	2, 2, 2, 2
29	$Pbcn$	Si, Ti, Ge, Zr, Hf, Sn	2, 2, 2, 2, 2, 2
30	$Pnma$	Ti, Ge	2, 2
31	$C2/m$	Ti	2
32	$Pca2_1$	Zr, Hf, (Pb)	2, 2, 2
33	$P4_2/nmc$	Zr, Hf	2, 2
34	$Fm\bar{3}m$	Zr, Ce, Hf	3, 3, 3
35	$Pnma$	Zr, Hf	2, 2
36	$Pnnm$	Ti, Ge, Sn, Hf, Pb	2, 2, 2, 2, 2
37	$P3_221$	Si, Ge	2, 2
38	$C2/c$	Si	2
39	$P4_12_12$	Si, Ge	2, 2
40	$C2/c$	Si	3
41	$Pmmn$	V, Nb, Ta	2, 2, 2
42	$P2_1/m$	V	2
43	$C2/c$	V, Nb, Ta	2, 2, 2
44	$Ama2$	Cr	2
45	$Pnma$	Mo	2
46	Pc	Mo, W	1, 2
47	$P\bar{1}$	Mo, W	2, 2
48	$P2_1/n$	Mo, W	2, 2
49	$Pcnb$	Mo, W	2, 2

Table S4. PAW datasets and Hubbard *U* parameters.

Element	PAW core radii (Å)	Valence orbitals and electrons	Hubbard *U* (orbital, U_{eff} (eV))
Li	1.085	$(2s)^1$	-
Be	1.005	$(2s)^2$	-
O	0.804	$(2s)^2$, $(2p)^4$	-
Na	1.164	$(3s)^1$	-
Mg	1.058	$(3s)^2$	-
Al	1.005	$(3s)^2$, $(3p)^1$	-
Si	1.005	$(3s)^2$, $(3p)^2$	-
K	1.640	$(4s)^1$, $(3p)^6$	-
Ca	1.588	$(4s)^2$	-
Sc	1.588	$(4s)^1$, $(3d)^2$	-
Ti	1.482	$(4s)^1$, $(3d)^3$	d, 3
V	1.429	$(4s)^1$, $(3d)^4$	d, 3
Cr	1.323	$(4s)^1$, $(3d)^5$	d, 3
Cu	1.217	$(4s)^1$, $(3d)^{10}$	d, 5
Zn	1.217	$(4s)^2$, $(3d)^{10}$	d, 5
Ga	1.217	$(4s)^2$, $(4p)^1$, $(3d)^{10}$	-
Ge	1.217	$(4s)^2$, $(4p)^2$	-
As	1.111	$(4s)^2$, $(4p)^3$	-
Rb	1.323	$(4s)^2$, $(4p)^6$, $(5s)^1$	-
Sr	1.323	$(4s)^2$, $(4p)^6$, $(5s)^2$	-
Y	1.482	$(4s)^2$, $(4p)^6$, $(5s)^1$, $(4d)^2$	-
Zr	1.323	$(4s)^2$, $(4p)^6$, $(5s)^1$, $(4d)^3$	d, 3
Nb	1.455	$(4s)^2$, $(4p)^6$, $(5s)^1$, $(4d)^4$	d, 3
Mo	1.455	$(4s)^2$, $(4p)^6$, $(5s)^1$, $(4d)^5$	d, 3
Ag	1.323	$(5s)^1$, $(4d)^{10}$	d, 5
Cd	1.217	$(5s)^2$, $(4d)^{10}$	d, 5
In	1.640	$(5s)^2$, $(5p)^1$	-
Sn	1.588	$(5s)^2$, $(5p)^2$	-
Sb	1.217	$(5s)^2$, $(5p)^3$	-
Sc	1.588	$(3d)^2$, $(4s)^1$	-
Ba	1.482	$(5s)^2$, $(5p)^6$, $(6s)^2$	-
La	1.482	$(5s)^2$, $(5p)^6$, $(6s)^2$, $(5d)^1$	-
Ce	1.429	$(5s)^2$, $(5p)^6$, $(6s)^2$, $(5d)^1$, $(4f)^1$	f, 5
Hf	1.588	$(6s)^1$, $(5d)^3$	d, 3
Ta	1.535	$(6s)^1$, $(5d)^4$	d, 3
W	1.455	$(6s)^1$, $(5d)^5$	d, 3
Au	1.323	$(6s)^1$, $(5d)^{10}$	d, 3
Hg	1.323	$(6s)^2$, $(5d)^{10}$	-
Tl	1.323	$(6s)^2$, $(6p)^1$, $(5d)^{10}$	-
Pb	1.640	$(6s)^2$, $(6p)^2$	-
Bi	1.588	$(6s)^2$, $(6p)^3$	-

Table S5. Hyperparameters of the simple-ANN. N means the number of dimensions of input SOAPs. Since we considered up to three hidden layers, each square bracket has up to three numbers indicating the number of nodes on the corresponding hidden layers. In addition, we used the weight decay rate of the regularization term, 1e-4, 1e-3, 1e-2, 1e-1, and 0.0, resulting in 90 combinations.

Name	Parameters
Network1	[N]
Network2	[0.25×N]
Network3	[0.5×N]
Network4	[0.75×N]
Network5	[N,N]
Network6	[0.25×N, 0.25×N]
Network7	[0.5×N, 0.5×N]
Network8	[0.75×N, 0.75×N]
Network9	[0.75×N, 0.5×N]
Network10	[N, 0.75×N]
Network11	[N, N, N]
Network12	[0.25×N, 0.25×N, 0.25×N]
Network13	[0.5×N, 0.5×N, 0.5×N]
Network14	[0.75×N, 0.75×N, 0.75×N]
Network15	[N, 0.75×N, 0.5×N]
Network16	[0.75×N, 0.75×N, 0.5×N]
Network17	[0.75×N, 0.5×N, 0.5×N]
Network18	[0.75×N, 0.5×N, 0.25×N]

Data S1 (separate file). Theoretical IPs and EAs of the 2,195 binary and 718 ternary oxide surfaces and related properties. The database contains the index, system (binary or ternary), chemical formula, space group number, Miller index, surface energy, IP, EA, and bandgap (bulk).

Data S2 (separate file). Supercells before and after structural optimization for the 2,195 binary and 718 ternary oxide surfaces. The number in the file name corresponds to the index in Data S1.
